# Supplementary material for: WAX INDUCER 1 Regulates β-Diketone Biosynthesis by Mediating Expression of the Cer-cqu Gene Cluster in Barley
Source: Int J Mol Sci. 2023 Apr 5;24(7):6762. doi: 10.3390/ijms24076762 (PMC10095013; doi:10.3390/ijms24076762)
Supplement: Supplementary file 1 [file ijms-24-06762-s001.zip › Supplementary Materials Figures.pdf]

Supporting Information figures

Article title: WAX INDUCER 1 regulates β-diketone biosynthesis by mediating expression of the *Cer-cqu* gene cluster in barley

The following Supporting Information is available for this article:

Figure S1. Developmental progress of the *win1* KO isogenic lines.

Figure S2. Dominant/recessive test.

Figure S3. The pattern of wax accumulation at all examined surfaces.

Figure S4. Chemical composition of cuticle wax in reproductive shoots of wild-type (WT) and *win1* knockout (KO) plants after derivatization.

Figure S5. Total ion chromatograms of wax samples.

Figure S6. Mass spectra of β-diketones.

Figure S7. Fragment of the chromatogram reconstruction for *win1* KO stem wax sample by the characteristic ion for alkylresorcinols (*m/z* 138) and mass spectra of some alkylresorcinols.

Figure S8. Verification of the RNA-seq results.

Figure S9. Amino acid sequence comparison between ScARS and putative HvARS.

Figure S1. Developmental progress of the *win1* KO isogenic lines.

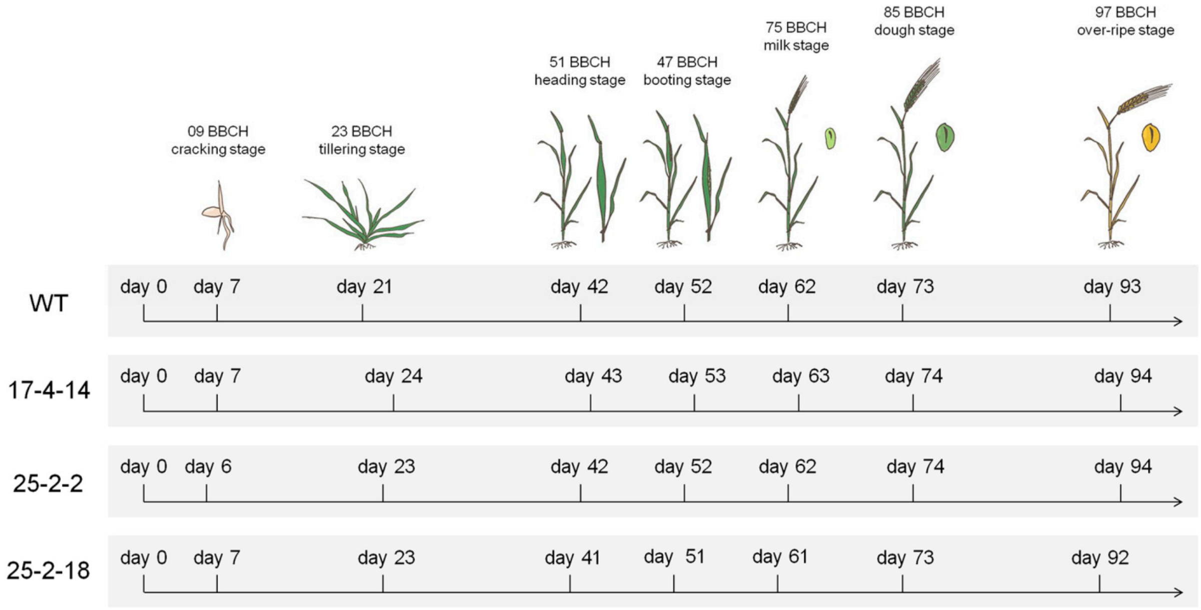

Figure S2. Dominant/recessive test. (a) Scheme of crossing. (b) *WIN1* target site Sanger chromatograms of parents and F1 hybrid, protospacer adjacent motif (PAM) is shown in red bar.

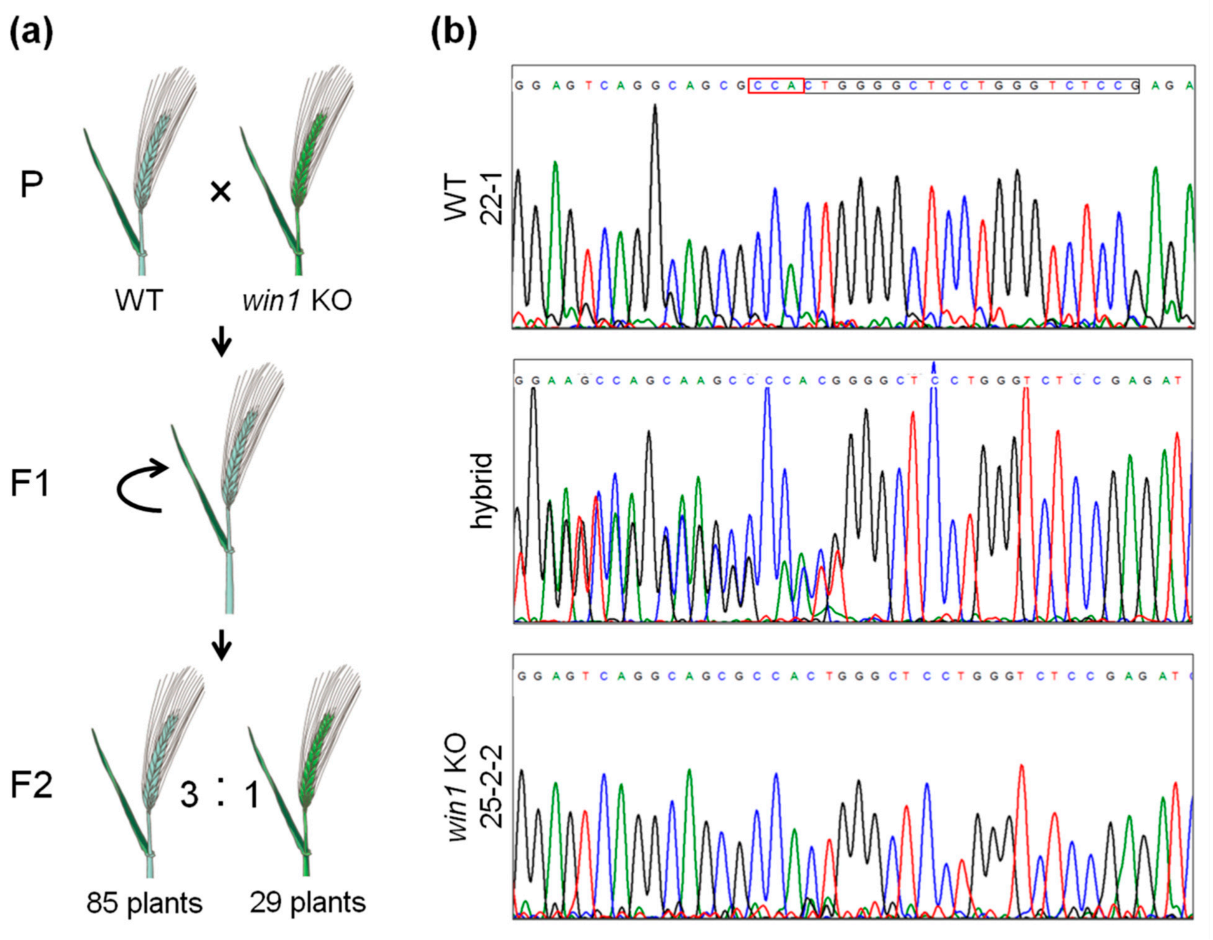

Figure S3. The pattern of wax accumulation at all examined surfaces. WT – wild type, *win1* KO – *win1* knockout, BBCH code is given according to Zadoks *et al.* (1974)

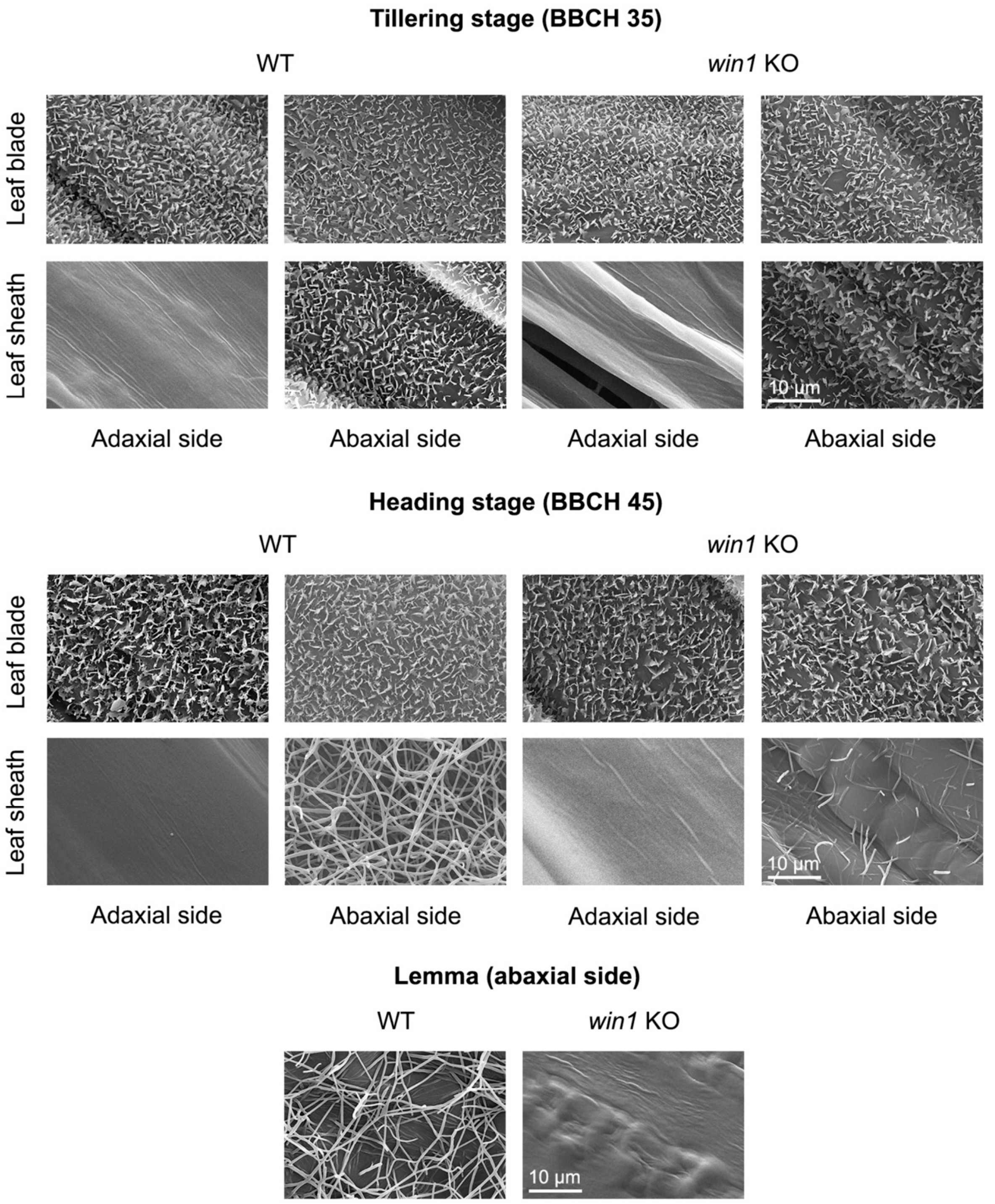

Figure S4. Chemical composition of cuticle wax in reproductive shoots of wild-type (WT) and *win1* knockout (KO) plants after derivatization, lb – leaf blade, st – stem, FAME – fatty acid methyl esters, HC – hydrocarbons, Ald – aldehydes, Alk – alkan-1-ols or alkan-2-ols, β-diketone - hentriacontane-14,16-dione, OH-β-diketone - 25-hydroxy-hentriacontane-14,16-dione, AR-1-17 – 3-methoxy-5-heptadecylphenol; AR-1-19 – 3-methoxy-5-nonadecylphenol; MAR-2-19 – 2-methyl-5-nonadecyl-1,3-benzenediol; MAR-2-21 – 2-methyl-5-heneicosyl-1,3-benzenediol, numbers in compound codes correspond to carbon atom numbers and positions of groups, asterisks show significant differences (* - p value <0.05).

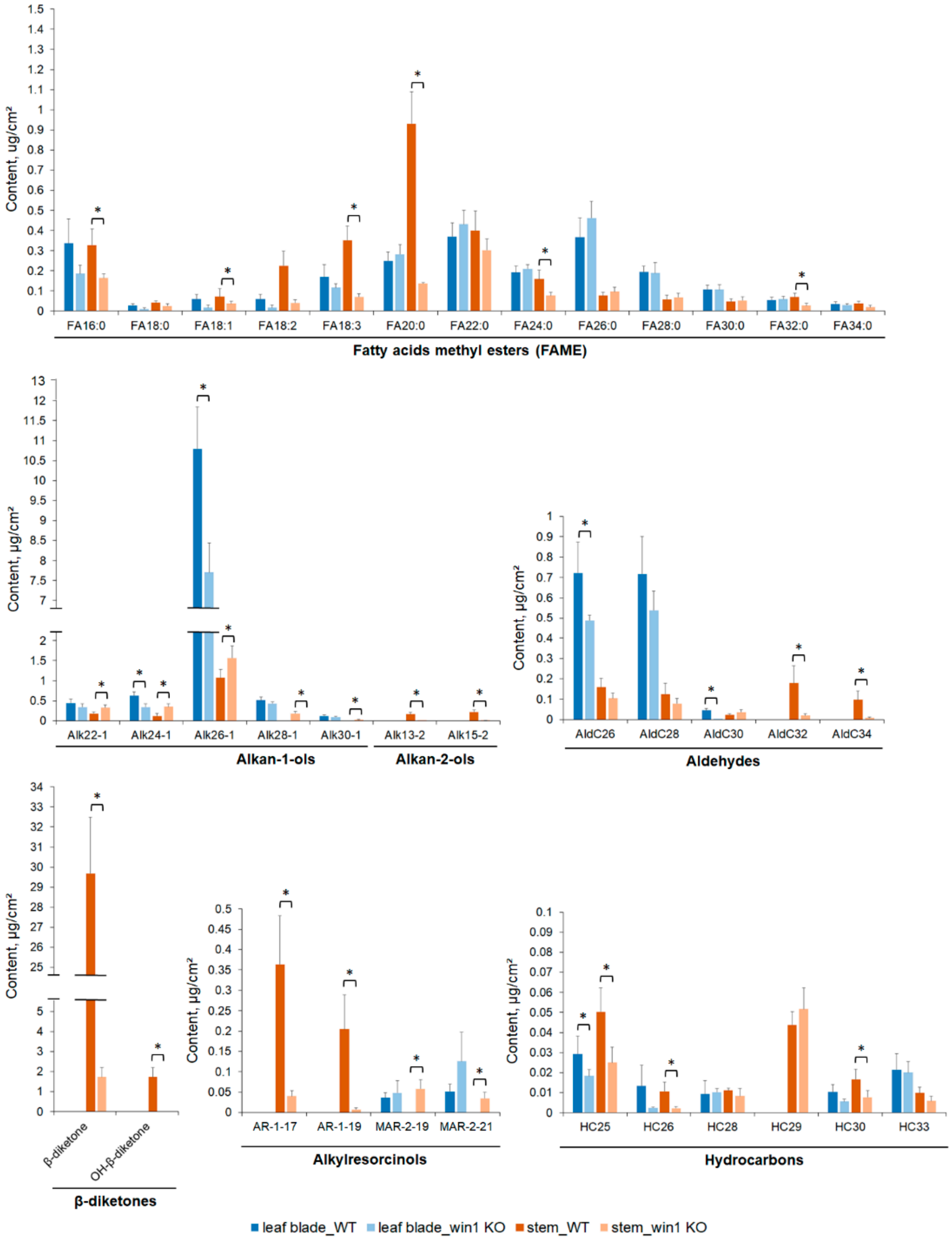

Content, ug/cm²
FA16:0
FA18:0
FA18:1
FA18:2
FA18:3
FA20:0
FA22:0
FA24:0
FA26:0
FA28:0
FA30:0
FA32:0
FA34:0
Fatty acids methyl esters (FAME)
Content, µg/cm²
Alk22-1
Alk24-1
Alk26-1
Alk28-1
Alk30-1
Alk13-2
Alk15-2
Alkan-1-ols
Alkan-2-ols
Content, µg/cm²
AldC26
AldC28
AldC30
AldC32
AldC34
Aldehydes
Content, µg/cm²
β-diketone
OH-β-diketone
β-diketones
Content, µg/cm²
AR-1-17
AR-1-19
MAR-2-19
MAR-2-21
Alkylresorcinols
Content, µg/cm²
HC25
HC26
HC28
HC29
HC30
HC33
Hydrocarbons
leaf blade_WT
leaf blade_win1 KO
stem_WT
stem_win1 KO

Figure S5. Total ion chromatograms of wax samples. (a) Wild-type leaf blades (b) Wild-type stems (c) *win1* knockout leaf blades (d) *win1* knockout stems. The numbering is shown with major peaks only. The numbering of the peaks corresponds to the numbering in the table S6.

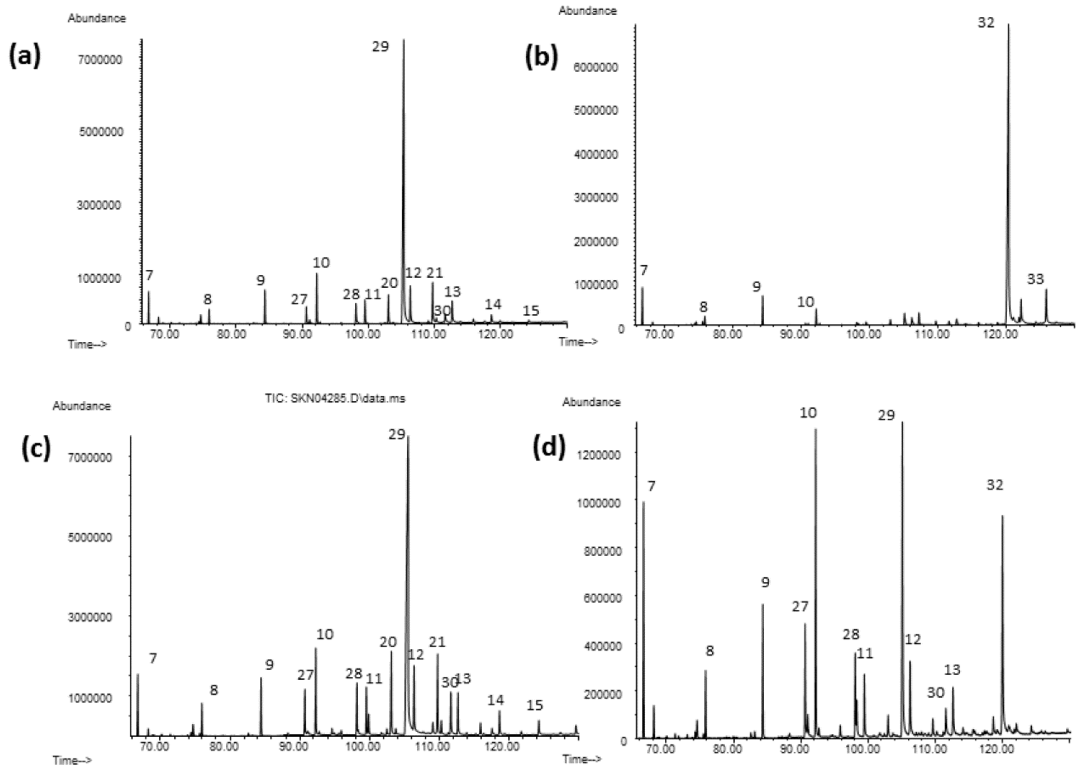

Figure S6. Mass spectra of β-diketones. (a) Nonacosane-14,16-dione (M436) (b) Hentriacontane-14,16-dione (M464) (c) Tritriacontane-16,18-dione (M492).

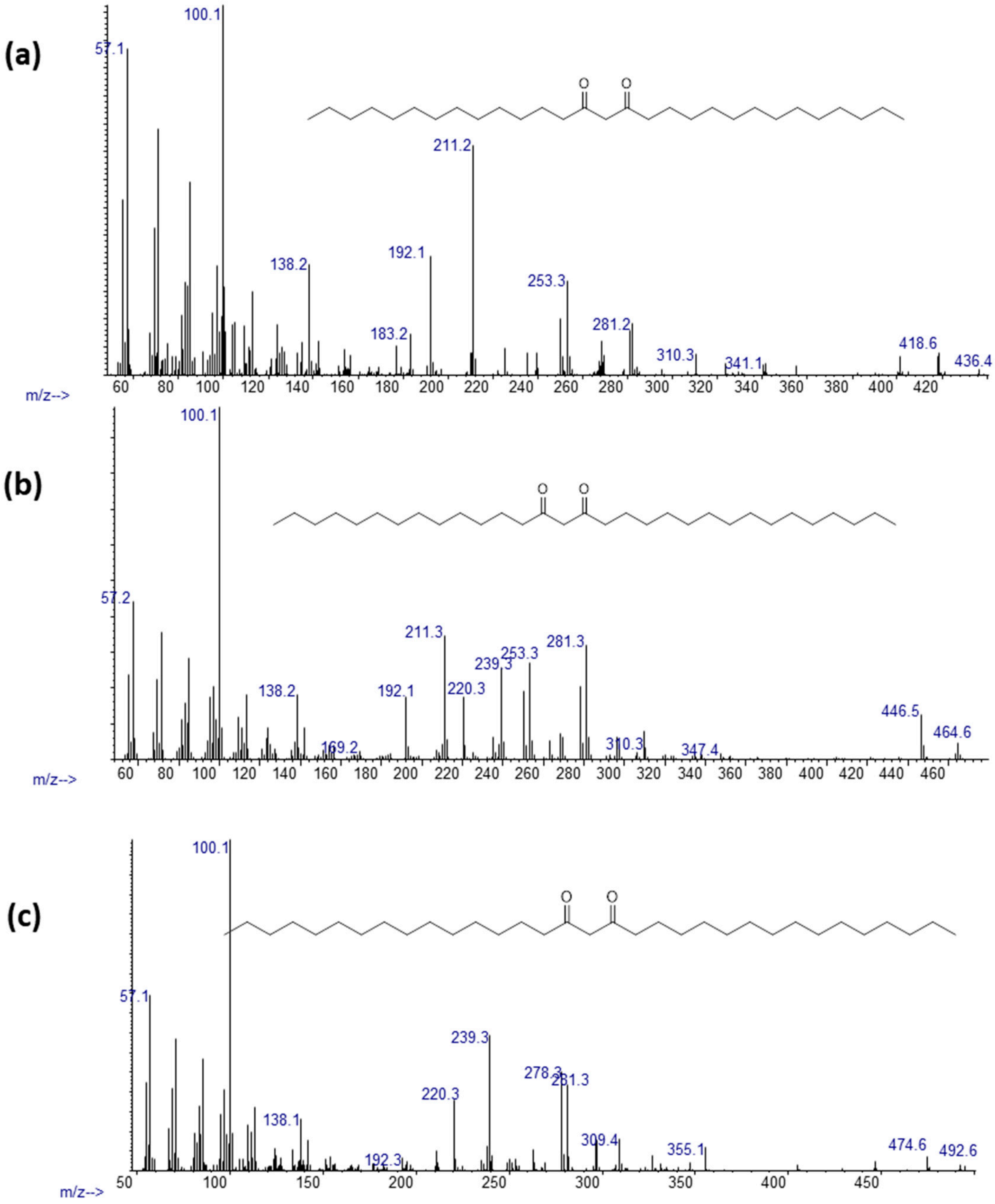

Figure S7. (a) Fragment of the chromatogram reconstruction for *win1* KO stem wax sample by the characteristic ion for alkylresorcinols (*m/z* 138) and mass spectra of some alkylresorcinols AR-1-15 – 3-Methoxy-5-pentadecylphenol; AR-1-17 – 3-methoxy-5-heptadecylphenol; AR-1-19 – 3-methoxy-5-nonadecylphenol; AR-1-21 – 3-methoxy-5-heneicosylphenol; MAR-2-19 – 2-methyl-5-nonadecyl-1,3-benzenediol; MAR-2-21 – 2-methyl-5-heneicosyl-1,3-benzenediol; MAR-2-23 – 2-methyl-5-tricosyl-1,3-benzenediol; (b) mass spectrum of AR-1-15, (c) mass spectrum of AR-1-19, (d) mass spectrum of MAR-2-21, (e) mass spectrum of MAR-2-23.

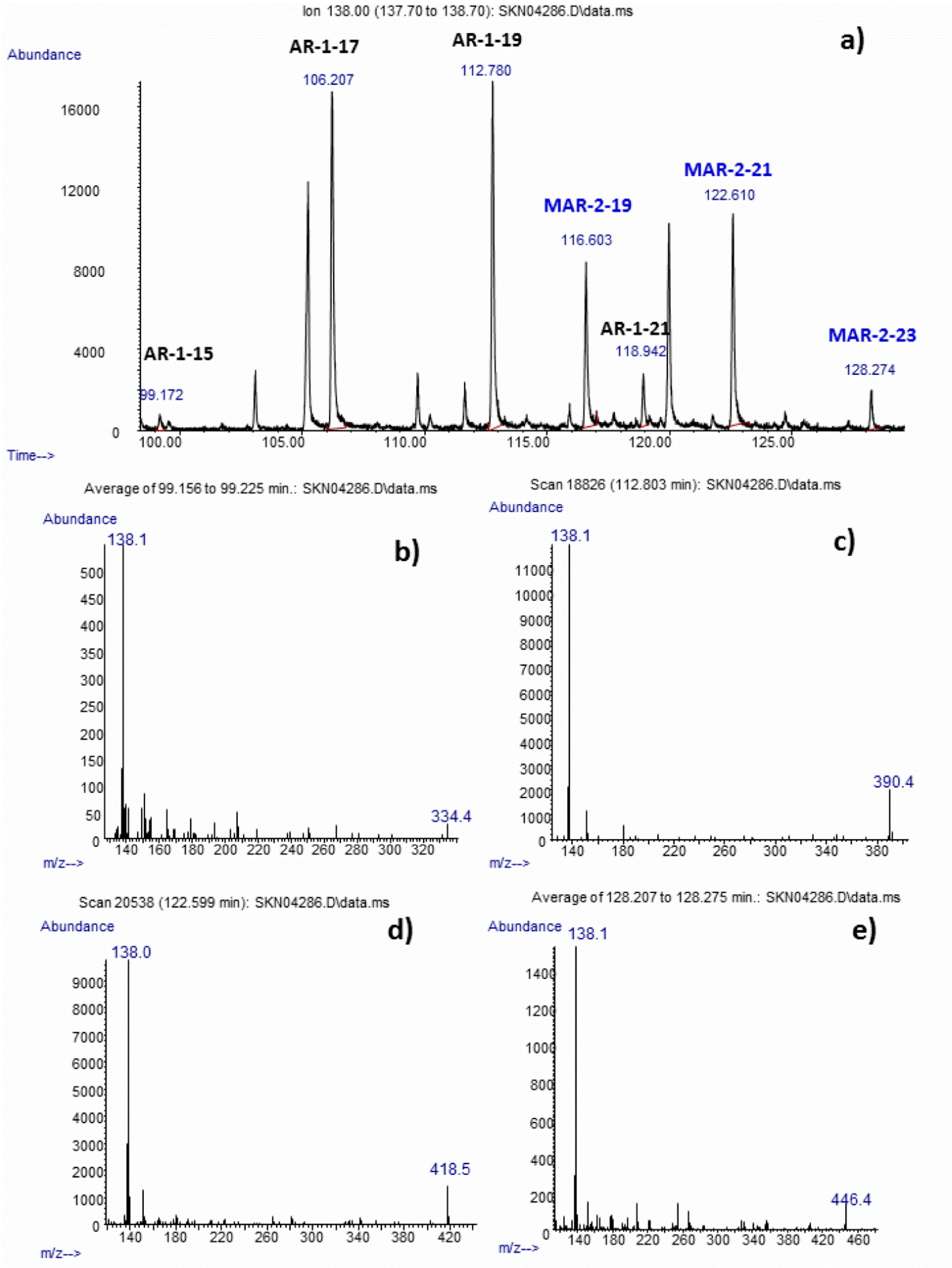
Ion 138.00 (137.70 to 138.70): SKN04286.D\data.ms
a)
Abundance
AR-1-15
99.172
AR-1-17
106.207
AR-1-19
112.780
MAR-2-19
116.603
AR-1-21
118.942
MAR-2-21
122.610
MAR-2-23
128.274
Time-->
Average of 99.156 to 99.225 min.: SKN04286.D\data.ms
b)
138.1
334.4
m/z-->
Scan 18826 (112.803 min): SKN04286.D\data.ms
c)
138.1
390.4
Scan 20538 (122.599 min): SKN04286.D\data.ms
d)
138.0
418.5
Average of 128.207 to 128.275 min.: SKN04286.D\data.ms
e)
138.1
446.4

Figure S8. Verification of the RNA-seq results. qRT-PCR results are shown by bars, RNA-seq results by lines, related to Figure 3. Standard deviations are shown for each case. lb_win1, lb_wt – leaf blade wild-type and *win1* KO respectively; ls_wt, ls_win1 – leaf sheath wild-type and *win1* KO respectively.

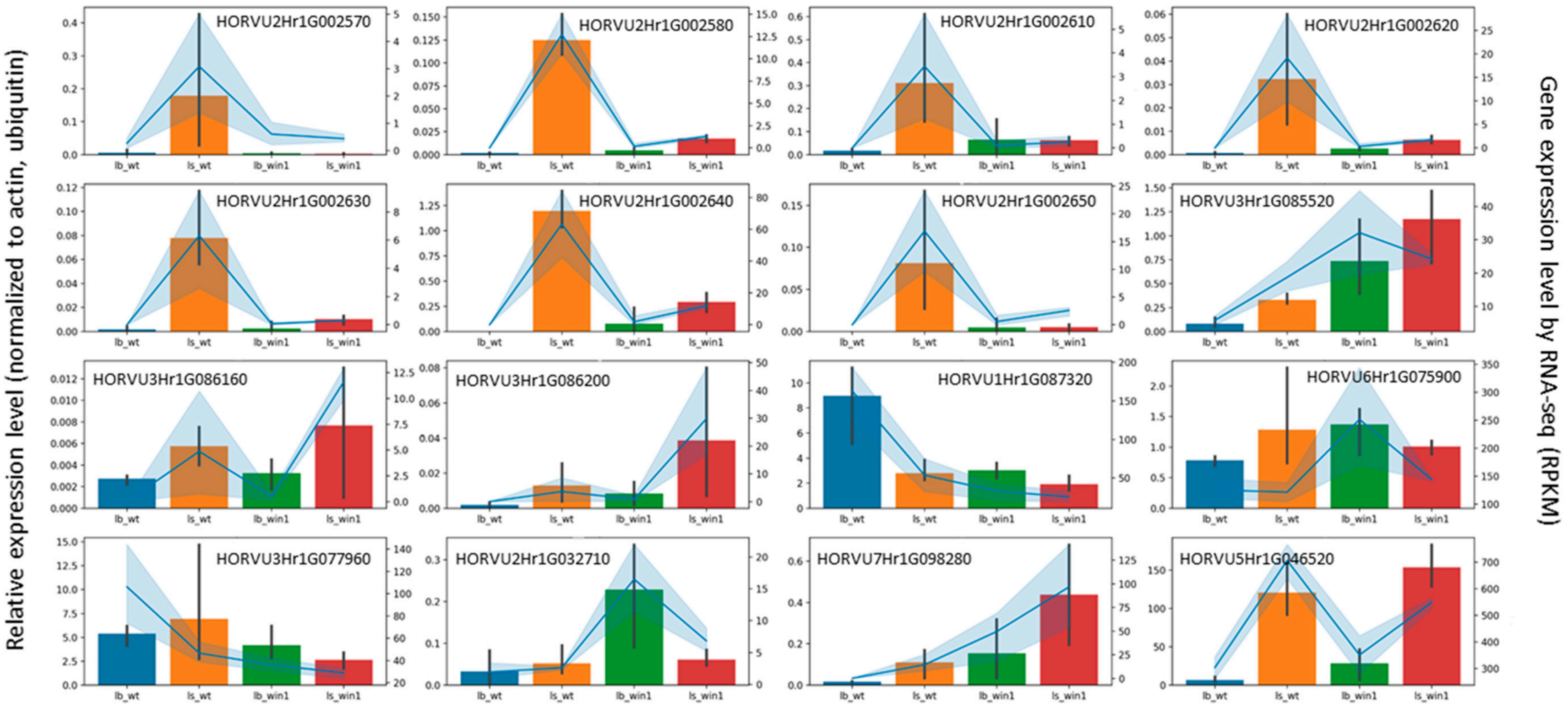

Figure S9. Amino acid sequence comparison between ScARS and putative HvARS.

```
HvARS      ------------------MGMRQHGEGPAAMLAIGTANPTNL-LPQDVFADNFFRMTKSD 41
ScARS      MGSIGTTNGNGIGHGSAAVARRQHAEGPAAMLGIGTANPTGVEVPQNIFAENLFRVTKSD 60
                             :. ***.*******.*******.: :**::**:*:**:****

HvARS      HLTQLKEKMNRICEKTGIEKRHFHLTEDTLGVHPEFLDRDLPSLDARIEMAATAVPKLAQ 101
ScARS      HLTELQLKLTRICEKTGIDKRHFHLTEETLAAHPELYDHEAPSLDNRIAMTVDAVPKLAQ 120
           ***:*: *:.********:********:**..***: *:: **** ** *:. *******

HvARS      SAATKAIAEWGRPATDITHLVFSTYSAWEAPSADLKLATLLGLPPTVCRTILSLHGCYGG 161
ScARS      CAAAKAIAEWGRPASEITHLVFSTYSAWGAPSADLRLAALLGLRPTVSRTILSLHGCYGG 180
           .**:**********::************ ******:**:**** ***.************

HvARS      GRALHLAKELAENNRGARVLVACAETTLVCLGSPDGANLVGYALFGDGAGAVIVGAGPFG 221
ScARS      GRALGLARELAENNRGARVLVACAEITLVCFGGPDGGNLVGHALFGDGAGAVIVGAGPFR 240
           **** **:***************** ****:*.***.****:*****************

HvARS      EGE-RPLFEMVSATQTTIPRTEHVLGMQATAGGIDFHLAIQVPMLIGQNVERCLLDAFGD 280
ScARS      DGEQSPIFEMVHATQTTVPKTEHVLGMQVSGSGVDFHLAIQVPTLIGQNVERCLLDAFRG 300
           :**  *:**** *****:*:********.:..*:********* ************** .

HvARS      D------------------APCDWNELFWAVHPGGRPILDNIDTVLKLDTGKLAASRHVL 322
ScARS      GDDGGDDDDDGAHLPSPLSGNGKWNDLFWAVHPGGRPILDNIDKVLQLEPEKLGASRHVL 360
           .                  .  .**:*****************.**:*:  **.******

HvARS      REYGNMSGATIVFVLDELRRRRKEEDNGHLLPEWGAMLAFGPGITIETMVLRSPR      377
ScARS      REYGNMSGATIVFVLDELRRRR------SLLPEWGAMLAFGPGVTIETMVLRCPR      409
           **********************       **************:********.**
```
