# Supplementary material for: WAX INDUCER 1 Regulates β-Diketone Biosynthesis by Mediating Expression of the Cer-cqu Gene Cluster in Barley
Source: Int J Mol Sci. 2023 Apr 5;24(7):6762. doi: 10.3390/ijms24076762 (PMC10095013; doi:10.3390/ijms24076762)
Supplement: Supplementary file 1 [file ijms-24-06762-s001.zip › Supplementary Materials Methods.pdf]

Supporting Information methods

Article title: WAX INDUCER 1 regulates β-diketone biosynthesis by mediating expression of the *Cer-cqu* gene cluster in barley

The following Supporting Information is available for this article:

Method S1. Gas chromatography–mass spectrometry.

Method S2. RNA-seq data analysis and verification.

Method S1. Gas chromatography–mass spectrometry.

We used a 6890N gas chromatograph and a mass-selective detector 5975N (Agilent Technologies, USA) at 70 eV with a gas flow rate of 1 ml/min He. Separation was achieved with an HP-5MS capillary column (30 m×0.25 mm, film thickness 0.25 µm). Two different temperature gradients were used as follows: for the identification and quantification of cuticular waxes, the temperature was initially held at 50 C for 2 min, raised to 280 C at a rate of 10 C min-1, and held at 280 C for 40 min; for linear retention index calculations, the temperature was initially held at 50 °C for 2 min, raised to 310 C at a rate of 2 C min-1, and held at 310 C for 13 min. We used ChemStation software for the identification and quantification of hydrocarbons, ketones, aldehydes, alcohols, alkylresorcinols and fatty acid methyl esters. The relative quantity of compounds was determined by the mean area of the chromatographic peak among two replicate injections. Compounds were quantified in solution with an extract concentration of 0.5 mg/mL. Compounds were quantified using tetracosane for hydrocarbons, 1-docosanol for alcohols, ketones, alkylresorcinols, and methyl octadecanoate for fatty acid methyl esters, and aldehydes. The calculation of the β-diketone content was corrected for the partial conversion of 14,16-hentriacontanedione to fatty acids and ketones under derivatization conditions [41,42].

1-Tetradecanol (Sigma–Aldrich) was used as an internal standard. Supelco 37-Component FAME Mix (47885-U), 1-hexacosanol and 1-octacosanol (Sigma–Aldrich), and a Policosanol alcohol mixture (C22-OH C32-OH) [43] were used for metabolite identification. The GC qualitative standard AG5080-8716 was used to calculate linear retention indices (LRIs) [44,45].

Hydrocarbons, ketones, aldehydes, alcohols, alkylresorcinols and fatty acid methyl esters were identified using the NIST 14 MS spectral library, LIPID MAPS® Lipidomics Gateway (https://www.lipidmaps.org/resources/lipidweb), and the LipidWeb data on GC/MS analysis of long-chain aldehydes [46,47]. In addition, to increase the reliability of identification, we used the method of obtaining extract ion chromatograms for characteristic ions: m/z 71, 85 – for saturated hydrocarbons, m/z 71 – for ketones, m/z 100 – for diketones, m/z 83 – for alcohols, m/z 82 – for aldehydes, m/z 138 – for alkylresorcinols, and m/z 74, 87 – for saturated fatty acid methyl esters. Experimental and literature data on the linear retention indices were used for reliable identification of alkylresorcinols [48].

The wax esters C29, C31, C33 and C35 formed by alkane-2-ols and C18, C20 and C22 fatty acids were identified in cuticle wax extracts of WT stems by gas chromatography–mass spectrometry after diazomethane treatment [39] performed for free fatty acid derivatization by characteristic mass spectra [49].

The area of a few dozens of leaf blades from examined plants was measured (photos of leaf blades were taken using an A4 sheet of paper as background; the average area per leaf blade was calculated and used as a coefficient for amounts defined per one leaf blade.

The lengths and widths of stem samples were measured when samples were taken. The surface area of the stems was approximated according to the form of a cylinder, and the average stem surface area was then used as a coefficient for amounts defined per one stem sample.

Methods S. RNA-seq data analysis and verification.

FASTQC v. 0.11.9 was used to evaluate library quality (https://www.bioinformatics.babraham.ac.uk/projects/fastqc/). Filtering of libraries was performed using Trimmomatic software v. 0.39 [50] with the following parameters: 'LEADING: 20, TRAILING: 20, SLIDINGWINDOW: 4:20, and MINLEN: 50'. Filtered reads were then mapped to the barley genome assembly IBSC v.2 (release 47) from the Ensembl Plants database (http://plants.ensembl.org) using the DART tool v.1.4.2. [51] with default parameters. Next, the number of reads aligning to each gene was counted with the featureCounts function in the Subread software package [52] with the following options: -M (counting multimapping reads), -O (counting multioverlapping reads) and –primary (considering only primary alignments for multiply aligned reads). Finally, to assess library quality and search for outliers, principal component analysis (PCA) using the DESeq2 function plotPCA was conducted [53]. In total, 468,743,831 single-end 75-bp raw reads were obtained. The raw reads consisted of 35,624,531,156 bases. After filtering, 440,992,952 (94%) reads were retained, consisting of 33,229,354,577 bases, with a mean GC content equal to 52.75% and a Q20 proportion over 99% in each sample. On average, 96.6% (77.94% uniquely) of reads were mapped to the reference barley genome using DART software.

Library statistics

Library	Library metrics			Read mapping		
	Number of raw reads	Number of clean reads	Percentage of retained reads	Uniquely mapped [%]	Multiple mapped [%]	Unmapped [%]
lb_wt.1	29555735	27867837	94.29	79.27	19.31	1.43
lb_wt.2	33890261	31954671	94.29	80.80	17.93	1.27
lb_wt.3	40456822	38310131	94.69	80.33	18.46	1.21
lb_wt.4	36705936	34626950	94.34	81.96	16.76	1.28
ls_wt.1	28325847	26674980	94.17	78.04	20.48	1.48
ls_wt.3	27867835	26213418	94.06	74.53	23.80	1.67
ls_wt.3	25793367	24112000	93.48	74.40	24.36	1.25
ls_wt.4	26834134	25317520	94.35	79.60	19.19	1.21
lb_win1.1	30903027	28823942	93.27	80.27	18.42	1.31
lb_win1.2	24590170	23048655	93.73	79.61	19.06	1.33
lb_win1.3	25863048	24250782	93.77	73.14	24.95	1.92
lb_win1.4	30644745	28858131	94.17	75.22	23.77	1.02
ls_win1.1	27448119	25782633	93.93	79.95	18.77	1.28
ls_win1.2	25608225	23868851	93.21	76.73	21.99	1.28
ls_win1.3	19392434	18372810	94.74	77.60	21.13	1.26
ls_win1.4	34864126	32909641	94.39	75.55	22.78	1.67

lb_win1, lb_wt – flag leaf blade wild-type and *win1*; ls_wt vs ls_win1 – flag leaf sheath wild-type and *win1*; 1-4 – number of library.

Based on the obtained expression data, a PCA was conducted. Libraries from the same conditions were grouped into easily separable clusters, except for lb_win1.3. This library was deemed an outlier and thus was excluded from further analysis.

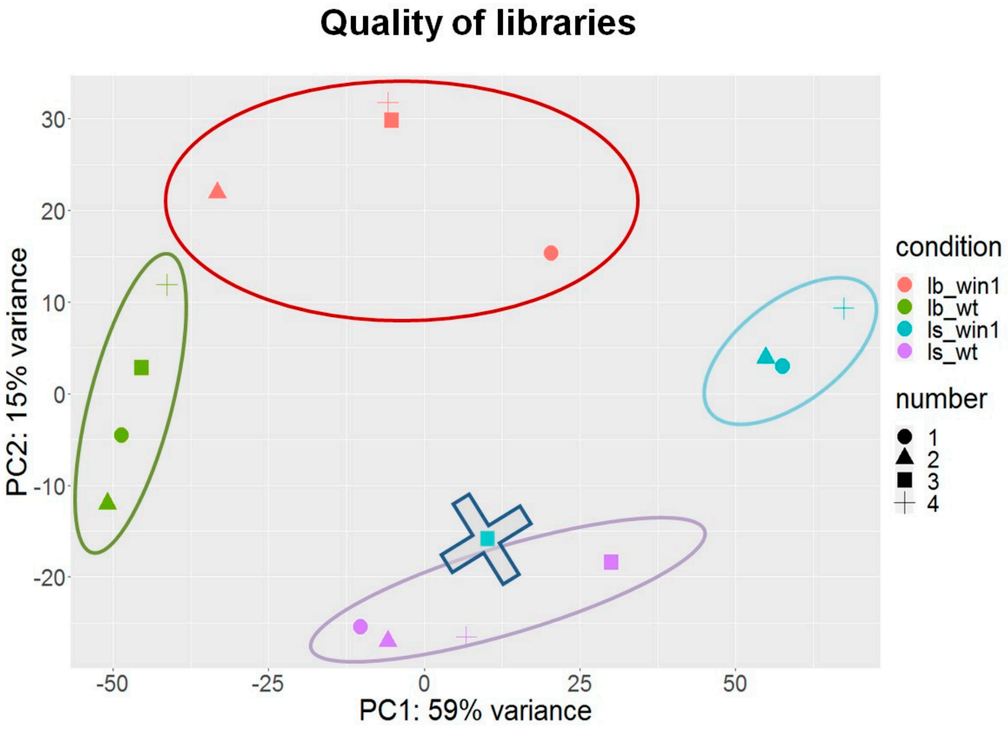

Principal component analysis (PCA) of libraries. lb_win1 – *win1* KO leaf blade, lb_wt – wild type leaf blade; ls_win1 – *win1* KO leaf sheath, ls_wt – wild type leaf sheath; 1-4 – number of library. Cross marks excluded library.

A differentially expressed gene search was performed using the edgeR software package for R [54]. Genes with low expression were eliminated using the 'filterByExpr' function. Counts per million base pair (CPM)-normalized reads are listed in the Supplemental Dataset S3. The generalized linear model (GLM) approach was used to detect differential expression between samples. Genes with FDR < 0.05 and $|logFC| > 2$ were considered differentially expressed. The following comparisons between samples were made: leaf blade of *win1* mutant against leaf blade of wild-type; leaf sheath of *win1* mutant against leaf sheath of wild-type; leaf blade of *win1* mutant against leaf sheath of *win1* mutant; and leaf blade of wild-type against leaf sheath of wild-type. Sixteen genes with different patterns of expression were chosen to perform qRT–PCR verification of the RNA-seq data. qRT–PCR was performed in a QuantStudio 5 (Applied Biosystems, http://www.lifetechnologies.com) based on the SYNTOL SYBRGreen I kit (Syntol, Moscow, Russia) in a 15-µL reaction mixture. The number of amplification cycles and the

annealing temperature were optimized for each primer pair (see Table S1). Each sample was run in three technical replications. Gene expression levels were calculated using the relative standard curve method and normalized against the geometric mean of *actin* and *ubiquitin* gene expression, with primers suggested by von Zitzewitz et al., 2005 and Himi et al., 2005, respectively [55,56].
