# Supplementary material for: WAX INDUCER 1 Regulates β-Diketone Biosynthesis by Mediating Expression of the Cer-cqu Gene Cluster in Barley
Source: Int J Mol Sci. 2023 Apr 5;24(7):6762. doi: 10.3390/ijms24076762 (PMC10095013; doi:10.3390/ijms24076762)
Supplement: Supplementary file 1 [file ijms-24-06762-s001.zip › Supporting_information_tables_corr.pdf]

Supporting Information tables

Article title: WAX INDUCER 1 regulates β-diketone biosynthesis by medi-ating expression of the *Cer-cqu* gene cluster in barley

The following Supporting Information is available for this article:

Table S1. General phenotyping of *win1* KO lines as compared to the wild-type.

Table S2. Chemical composition of cuticular waxes detected on barley leaf blades and stems after derivatization.

Table S3. Differential expression of all barley genes in all four performed comparisons (see separate file).

Table S4. Information about *Cer-cqu* cluster and related genes located at chromosome 2H (see separate file).

Table S5. Barley homologues of *Arabidopsis thaliana* genes involved in wax biosynthesis (see separate file).

Table S6. Expression (rpkm-normalized counts) of putative WIN1-regulated genes (see separate file).

Table S7. Primers used in the study.

Table S8. Counts per million mapped read (CPM)-normalized counts of gene expression obtained using the edgeR software package (see separate file).

Table S9. Raw counts of gene expression obtained using DART mapper and featureCounts function of the Subread package (see separate file).

Table S1. General phenotyping of win1 KO lines as compared to the wild-type with standard deviations being indicated.

Trait	Control line	Win1 KO lines		
	WT	17-4-14	25-2-2	25-2-18
Spikelet number per spike	11.8 ± 0.6	12.8 ± 1.0	12.4 ± 0.8	12 ± 1.3
Spike length, cm	8.7 ± 0.9	8.7 ± 0.3	8.6 ± 0.9	8.8 ± 0.9
Grain number per spike	14 ± 4.6	18.6 ± 3.2	17.2 ± 3	16.8 ± 4
Grain number per plant	111.3 ± 33.8	139.9 ± 48.8	122.4 ± 24.3	100.3 ± 32.4
Grain weight per spike, g	0.66 ± 0.2	0.6 ± 0.24	0.78 ± 0.13	0.76 ± 0.17
Grain weight per plant, g	4.12 ± 0.81	4.64 ± 1.87	5.37 ± 1.36	4.44 ± 1.63
1000-grain weight, g	38.1 ± 5.4	32.7 ± 4.2	43.6 ± 4.7	43.5 ± 4.5
Plant height, cm	62.5 ± 4.6	65.1 ± 4.2	66.5 ± 3.1	64.8 ± 4.4
Total number of tillers	9.1 ± 2.7	9.9 ± 2.6	8.6 ± 1.8	7.4 ± 3.1
Number of fertile tillers	7.9 ± 2.3	8 ± 2.7	7.9 ± 1.8	6.6 ± 2.5

Table S2. Chemical composition of cuticular waxes detected on barley leaf blades and stems after derivatization.

	IUPAC Name	Linear retention index (LRI), exp.	Content [µg/cm^2]			
			Leaf blade		Stem	
			Control n=4	Mutant n=4	Control n=4	Mutant n=4
			Hydrocarbons			
1.	Pentacosane	2500	0.029±0.009	0.018±0.003	0.05±0.01	0.025±0.008
2.	Hexacosane	2600	0.013±0.010	0.002±0.001	0.011±0.005	0.002±0.001
3.	Octacosane	2800	0.009±0.007	0.010±0.002	0.011±0.001	0.008±0.004
4.	Nonacosane	2900	a	a	0.044±0.006	0.052±0.010
5.	Triacontane	3100	0.010±0.004	0.006±0.004	0.017±0.005	0.008±0.003
6.	Tritriacontane	3300	0.021±0.008	0.020±0.005	0.010±0.003	0.006±0.002
			Saturated fatty acids methyl esters			
7.	Hexadecanoic acid	1928	0.34±0.12	0.19±0.04	0.33±0.08	0.16±0.02
8.	Octadecanoic acid	2128	0.17±0.06	0.12±0.02	0.35±0.07	0.07±0.01
9.	Eicosanoic acid	2329	0.25±0.04	0.28±0.05	0.93±0.16	0.14±0.01
10.	Docosanoic acid	2532	0.37±0.07	0.43±0.07	0.40±0.10	0.30±0.06
11.	Tetracosanoic acid	2731	0.19±0.03	0.21±0.02	0.16±10.04	0.08±0.02
12.	Hexacosanoic acid	2936	0.37±0.09	0.46±0.08	0.08±0.01	0.10±0.02
13.	Octacosanoic acid	3138	0.19±0.03	0.19±0.05	0.06±0.02	0.07±0.02
14.	Triacontanoic acid	3342	0.11±0.02	0.11±0.02	0.04±0.01	0.05±0.02
15.	Dotriacontanoic acid	3542	0.06±0.01	0.06±0.01	0.07±0.02	0.03±0.01
16.	Tetratriacontanoic acid	3741	0.04±0.01	0.03±0.01	0.04±0.01	0.02±0.01
			Unsaturated fatty acids methyl esters			
17.	Octadecenoic acid	2098	0.06±0.02	0.02±0.01	0.22±0.07	0.04±0.02
18.	Octadecadienoic acid	2092	0.03±0.01	0.01±0.01	0.04±0.01	0.03±0.01
19.	Octadecatrienoic acid	2098	0.06±0.02	0.02±0.01	0.07±0.04	0.04±0.01
			Aldehydes			
20.	Hexacosanal	2833	0.72±0.15	0.49±0.03	0.16±0.04	0.10±0.03
21.	Octacosanal	3043	0.72±0.18	0.54±0.10	0.12±0.05	0.08±0.02
22.	Triacontanal	3244	0.04±0.01	<0.01	0.023±0.006	0.036±0.014
23.	Dotriacontanal	3452	<0.01	<0.01	0.18±0.09	0.02±0.01
24.	Tetratriacontanal	3652	<0.01	<0.01	0.10±0.04	0.007±0.005
			Alkan-2-ols			
25.	Tridecan-2-ol	1503	b	b	0.16±0.05	0.002±0.001

26.	Pentadecan-2-ol	1704	b	b	0.22±0.05	0.007±0.001
	Alkan-1-ols					
27.	Docosan-1-ol	2489	0.44±0.11	0.34±0.07	0.18±0.04	0.33±0.07
28.	Tetracosan-1-ol	2694	0.63±0.09	0.34±0.07	0.12±0.06	0.35±0.07
29.	Hexacosan-1-ol	2901	10.8±1.1	7.7±0.7	1.1±0.2	1.6±0.3
30.	Octacosan-1-ol	3103	0.51±0.09	0.43±0.04	b	0.18±0.05
31.	Triacontan-1-ol	3309	0.12±0.02	0.09±0.02	b	0.03±0.01
	β-diketones					
32.	Hentriacontane-14,16-dione	3395	b	b	29.7±2.8	1.7±0.5
33.	25-Hydroxy-hentriacontane-14,16-dione	3586	b	b	1.7±0.5	b
	Alkylresorcinols					
34.	3-Methoxy-5-pentadecylphenol	2718	b	b	a	a
35.	3-Methoxy-5-heptadecylphenol	2935	b	b	0.36±0.12	0.04±0.01
36.	3-Methoxy-5-nonadecylphenol	3144	b	b	0.20±0.08	0.007±0.004
37.	3-Methoxy-5- heneicosylphenol	3351	b	b	c	c
38.	2-Methyl-5-nonadecyl-1,3-benzenediol	3268	0.04±0.01	0.05±0.03	b	0.06±0.02
39.	2-Methyl-5-heneicosyl-1,3-benzenediol	3481	0.05±0.02	0.13±0.07	b	0.03±0.01
40.	2-Methyl-5-tricosyl-1,3-benzenediol	3687	b	0.02±0.01	b	0.01±0.01

[a] it was impossible to determine the individual contents due to overlapping peaks of the corresponding alcohols, [b] not found, [c] was identified below the limit of determination

Table S3. Differential expression of all barley genes in all four performed comparisons. Sheets: lb_win1-lb_wt: comparison between leaf blade of *win1* knockout genotype and leaf blade of wild type plants; ls_win1-ls_wt: comparison between leaf sheath of *win1* knockout genotype and leaf sheath of wild-type plants; lb_win1-ls_win1: comparison between leaf blade of *win1* knockout plants and leaf sheath of *win1* knockout plants; lb_wt-ls_wt: comparison between leaf blade and leaf sheath of wild-type plants.

Table S4. Information about the *Cer-cqu* cluster and related genes located on chromosome 2H.

Table S5. Barley homologues of *Arabidopsis thaliana* genes involved in wax biosynthesis.

Table S6. Expression (rpkm-normalized counts) of putative WIN1-regulated genes.

Table S7. Hexamers enriched in promoters of WIN1-related genes. Sheets: leaf sheath wt upreg – hexamers enriched in promoters of genes up-regulated in wild-type leaf sheaths, leaf sheath wt downreg – hexamers enriched in promoters of genes down-regulated in wild-type leaf sheaths.

Table S7. Primers used in the study.

Gene id	Primer	Sequence	Amplicon length [b]	Optimal annealing temperature [°C]
Primers used for target loci sequencing				
HORVU6Hr1G038120 (*HvWIN1*)	Win1_F	CGTACTAAGTGACTCCAGCAG	450	60
	Win1_R	TAAACCGGCGAAGAAACCAC		
HORVU6Hr1G085850	6H_gene_F	GAGAAGTCCGAGTCTAGCAG	960	59
	6H_gene_R	AAACGGGCTAAACAAAGATGTC		
HORVU7Hr1G029870	7H_gene_F	TTTGCCAACTAAACGTGTCACC	530	62
	7H_gene_R	CAATCGCAGAGCCTTTCGAC		
HORVU7Hr1G089930 (*HvNUD*)	Hv_Nud_1exF	AGCGTAGGTGTATACGCATCCG	1000	58
	Hv_Nud_R2	CAATGTGGGACTTCTCGGTG		
Primers used for qRT-PCR				
HORVU2Hr1G002570	2570_F1	AGACGACCTGCTTGGGCAAATG	116	60
	2570_R1	CGTAACCAGCCGCGAAGAAAG		
HORVU2Hr1G002580	2580_F1	CGGGTTGCGGTTCTTTGTCAC	174	62.5
	2580_R1	GCCCTTTACTTTTGCGCGCTG		
HORVU2Hr1G002610	2610_F1	GGCCCCATAGCTTTCCTGCA	74	60

	2610_R1	GTTCCTTTCGGCACCTTCACG		
HORVU2Hr1G002620	2620_F1	TATCCCGTAGGTCACCCGTCA	117	60
	2620_R1	GGCCATCCTTGTACTTGCGG		
HORVU2Hr1G002630	2630_F1	TTGCCGGCGTGTTGGTTTACG	129	60
	2630_R1	CATGGTAGTGTGCGTAAGCGC		
HORVU2Hr1G002640	2640_F1	TCCGTCTCTCAAACCCTCGTG	102	60
	2640_R1	CAGTGTGGGCAGTTTGGTGTAA		
HORVU2Hr1G002650	2650_F1	GTGAAGGTGCCGAAAGGAACG	135	60
	2650_R1	GACACAGACCCTCGGCCC		
HORVU3Hr1G085520	5520_F1	CGCTCAACACGCACCTCTTC	89	60
	5520_R1	CGTGGTGTAGTTGGCGGTGTA		
HORVU3Hr1G086160	6160_F3	GGCCCCATCGTCATCCTCC	100	61
	6160_R3	CCTCCGTGTCCGCCTTCC		
HORVU3Hr1G086200	6200_F4	CCGCAGCCTCGCCACAAAAC	77	62
	6200_R4	TCTTGGGGCACGGGAGCG		
HORVU1Hr1G087320	7320_F1	GCTCACGCTCGACCAGGG	147	60
	7320_R1	TCGCCCTCCCCAGACGTC		
HORVU6Hr1G075900	5900_F1	CGGGAGGCGTTCGTGGAG	124	60
	5900_R1	TGGTGTTGACGGCGAGCTTG		
HORVU3Hr1G077960	7960_F1	GGCGCTGGTGCTGGATCAAAG	65	60
	7960_R1	CTCTTTCTTCCGTTGCGCTGC		
HORVU2Hr1G032710	2710_F1	AGCCAGCAAGAACCACCGAGAA	165	60
	2710_R1	CAGACTTGGATTGAGCCCTTGC		
HORVU7Hr1G098280	8280_F1	TCCGGGTTCCAGTCCAAGCA	70	60
	8280_R1	GCCGGGGACGAGCTTGAG		
HORVU5Hr1G046520	6520_F1	ACCACCACACCCACTCATCTG	162	60
	6520_R1	GAGTCCACCTGCCCGCAC		
Primers used for transgene analysis				
	Cas9_F	GACGATGACCTGGATAACCT	300	58
	Cas9_R	GATGAACTTGTAGAACTCCTCCT		58

Table S8. Counts per million mapped read (CPM)-normalized counts of gene expression, obtained using the edgeR software package, raw data. lb_win1, lb_wt – leaf blade wild-type and *win1* KO respectively; ls_wt, ls_win1 – leaf sheath wild-type and *win1* KO respectively. Numbers represent biological replicates.

Table S9. Raw counts of gene expression obtained using DART mapper and featureCounts function of the Subread package. lb_win1, lb_wt – leaf blade wild-type and *win1* KO respectively; ls_wt, ls_win1 – leaf sheath wild-type and *win1* KO respectively. Numbers represent biological replicates.
